# Comparison of Medial and Lateral Meniscus Root Tears

**DOI:** 10.1371/journal.pone.0141021

**Published:** 2015-10-21

**Authors:** Ji Hyun Koo, Sang-Hee Choi, Seung Ah Lee, Joon Ho Wang

**Affiliations:** 1 Department of Radiology, Health Promotion Center, Samsung Medical Center, Sungkyunkwan University School of Medicine, Seoul, Korea; 2 Department of Radiology, Samsung Medical Center, Sungkyunkwan University School of Medicine, Seoul, Korea; 3 Department of Physical Medicine and Rehabilitation, College of Medicine, Kyung Hee University, Seoul Korea; 4 Department of Orthopedic Surgery, Samsung Medical Center, Sungkyunkwan University School of Medicine, Seoul, Korea; Van Andel Institute, UNITED STATES

## Abstract

The meniscus root plays an essential role in maintaining the circumferential hoop tension and preventing meniscal displacement. Studies on meniscus root tears have investigated the relationship of osteoarthritis and an anterior cruciate ligament tear. However, few studies have directly compared the medial and lateral root tears. To assess the prevalence of meniscal extrusion and its relationship with clinical features in medial and lateral meniscus root tears, we performed a retrospective review of the magnetic resonance imaging (MRI) results of 42 knee patients who had meniscus posterior horn root tears and who had undergone arthroscopic operations. The presence of meniscal extrusion was evaluated and the exact extent was measured from the tibial margin. The results were correlated with arthroscopic findings. Clinical features including patients’ ages, joint abnormalities, and previous trauma histories were evaluated. Twenty-two patients had medial meniscus root tears (MMRTs) and twenty patients had lateral meniscus root tears (LMRTs). Meniscal extrusion was present in 18 MMRT patients and one LMRT patient. The mean extent of extrusion was 4.2mm (range, 0.6 to 7.8) in the MMRT group and 0.9mm (range, -1.9 to 3.4) in the LMRT group. Five patients with MMRT had a history of trauma, while 19 patients with LMRT had a history of trauma. Three patients with MMRT had anterior cruciate ligament (ACL) tears, while 19 patients with LMRT had ACL tears. The mean age of the patients was 52 years (range: 29–71 years) and 30 years (range: 14–62 years) in the MMRT and LMRT group, respectively. There was a significant correlation between a MMRT and meniscal extrusion (*p*<0.0001), and between an ACL tear and LMRT (*p*<0.0001). A history of trauma was significantly common in LMRT (*p*<0.0001). LMRT patients were significantly younger than MMRT patients (*p*<0.0001). Kellgren-Lawrence (K-L) grade differed significantly between MMRT and LMRT group (*p*<0.0001). Meniscal extrusion is common in patients with MMRTs. However, it is rare in patients with LMRTs, which are more commonly associated with a history of trauma and ACL tears.

## Introduction

The meniscus is a fibrocartilaginous structure of the knee joint that enables the even distribution of a weight-bearing load across the articular surfaces. The menisci bear between 40 to 70% of the load across the knee, and the rest of the load is transmitted directly to the articular cartilage. Therefore, there have been reports of a positive correlation between meniscal tears and premature chondromalacia or osteoarthritis. The meniscus has several other important biomechanical functions. It absorbs shocks, protecting the joint. It also keeps the joint lubricated and regulates the joint's movement to prevent over extension. Therefore, a meniscal tear alters the functioning of the knee joint and may contribute to articular instability [1–10].

Of the various types of meniscal tears, a meniscus root tear is defined as a tear in the periphery of the meniscus where the meniscus attaches to the central tibial plateau [6]. The meniscus root plays an essential role in maintaining circumferential hoop tension and preventing meniscal displacement during axial loading. There have been many studies on medial meniscus root tears, particularly on the relationship of the tear with medial femorotibial osteoarthritis through meniscus extrusion [2, 6–10]. In addition, some studies have investigated the relationship of lateral meniscus root tears with anterior cruciate ligament tears [11–15]. However, few studies have directly compared the medial and lateral root tears.

Therefore, the objective of our study was to determine whether or not the MRI findings differ between medial and lateral meniscus root tears in terms of the incidence of meniscal extrusion and concomitant abnormal findings. We also evaluated if there were differences in clinical features, such as age and a history of trauma between medial and lateral meniscal root tears.

## Materials and Methods

We obtained approval and a waiver of informed patient consent from our institutional review board (Samsung Medical Center Institutional Review Board, IRB file number: 2011-10-030) for this retrospective study. The study was also performed in compliance with the Health Insurance Portability and Accountability Act (HIPAA). The patient information and records were anonymized and de-identified prior to analysis. From January 2006 to December 2012, 53 patients were arthroscopically confirmed as having meniscus posterior horn root tears. The root tear was diagnosed when there was a tear within 1 cm of the posterior tibial attachment according to the arthroscopic findings. Complex tears with horizontal or flap tears were also included. However, 11 patients were excluded from the study due to the quality of their MRI images being too poor to evaluate the meniscus roots. Finally, 42 patients were included in the study. Twenty patients had lateral meniscus root tears, while 22 patients had medial meniscus root tears. There were 17 men and three women in the lateral meniscus root tear group (mean age: 30 years, range: 14–62 years). There were six men and 16 women in the medial meniscus root tear group (mean age: 52 years, range: 29–71 years).

### Magnetic resonance imaging

Magnetic resonance (MR) images were obtained by using a 3.0-T MRI scanner (Intera Achieva; Philips Medical Systems, Best, the Netherlands). The extremity coil was used in a neutral position. The turbo spin-echo proton-density-weighted images, the sagittal turbo spin-echo T2-weighted images, the axial fat-saturated turbo spin-echo proton-density-weighted images and the anterior cruciate ligament oblique fat-saturated turbo spin-echo proton-density-weighted images in the coronal and sagittal planes were acquired. The range of the repetition time and echo time varied (3000–4000/10–30 ms for the proton-density-weighted images and 3000–5000/100 ms for the T2-weighted images). The other imaging parameters were as follows: matrix: 220 x 247 (axial and coronal) and 304 x 301 (sagittal); field of view: 16 cm; slice thickness: 4 mm (axial and coronal planes) and 1.5 mm (sagittal plane); and number of acquisitions: 1.

### Image analysis

Two radiologists (S.H.C., J.H.K., with 24 and 7 years of experience in musculoskeletal MR imaging, respectively) reviewed the MRI of the patients using a picture archiving and communicating system (Centricity Radiology RA 1000; GE Healthcare, Chicago, Ill). Both readers were blinded to the arthroscopic results and original radiology reports. A consensus reading was achieved when both readers agreed that a meniscal tear was present or not present on an MRI exam.

A meniscal root tear was defined as a tear within 1 cm of the tibial attachment of the meniscus. We used several MRI signs to determine a meniscal root tear: first, linear high signal intensity perpendicular to the meniscus on the meniscal root in the axial plane (radial tear); second, a vertical linear defect on the meniscal root in the coronal plane (the truncation sign), and third, increased signal intensity on the meniscal root in the sagittal plane (the ghost meniscus sign) [16].

**Fig 1:**
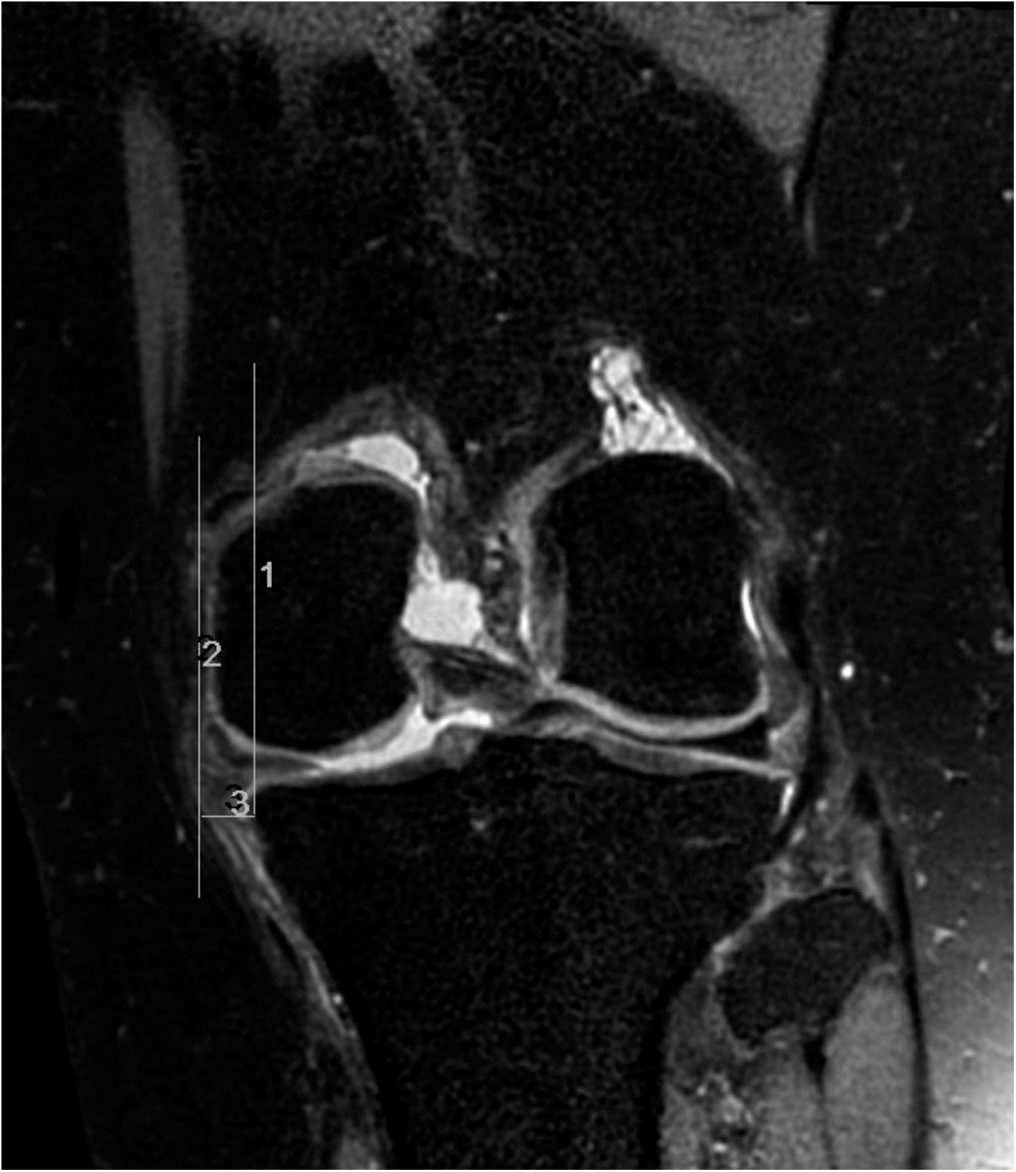
Size measurement of meniscal extrusion. Two vertical lines are drawn in the coronal plane. Line 1 is drawn at the tibial margin, and line 2 is drawn at the end of the extruded meniscus. Between the two vertical lines, the size of the extruded meniscus is measured as line 3.

In addition, we also looked for and evaluated meniscal extrusion with the criterion of extrusion greater than 3mm and we measured the exact extent of the medial margin of the proximal tibia in the coronal plane (Fig 1).

An anterior cruciate ligament or posterior cruciate ligament tear was diagnosed when discontinuity or focal or diffuse high signal intensity was present in the ligaments.

Knee joint x-rays were reviewed and the degree of osteoarthritis was evaluated using Kellgren-Lawrence (K-L) grade.

### Statistical analysis

Statistical analysis was performed using the SAS version 9.4 (SAS Institute, Cary, NC, USA). The t-test, Fisher's exact test and Chi-square tests were used to compare demographic data between the medial meniscus root tear and the lateral meniscus root tear groups. Cochran-Armitage trend test was used in the comparison of Kellgren-Lawrence grade. Chi-square test was used to determine whether meniscus extrusion was significantly more frequent in the medial meniscal root tear group than in the lateral meniscal root tear group. A statistically significant difference was considered to be present if the *p* value was less than 0.05.

## Results

In MMRT group, 16 patients showed increased signal intensity on the meniscal root in the sagittal plane (the ghost meniscus sign). Among them, coronal scan (the truncation sign) or axial scan (radial tear) also revealed meniscal root tear. In 6 patients, MMRTs were seen only in the coronal scan. In LMRT group, 17 patients showed the ghost meniscus sign in the sagittal plane and meniscal tears were also seen in coronal or axial scans in 8 out of 17 patients. In only 3 patients, LMRTs were seen in the coronal scan only.

Out of 22 patients (10 left knees, 12 right knees) who had medial meniscus posterior horn root tears (MMRTs), meniscal extrusion was present in 18 patients. The mean extent of the distance from the medial margin of the proximal tibia to the end of the extruded meniscus in the coronal plane was 4.2mm (range, 0.6 to 7.8). On the other hand, out of 20 patients (10 left knees, 10 right knees) who had lateral meniscus posterior horn root tears (LMRTs), meniscal extrusion was present in only one patient. The mean extent of the distance between the tibial margin and the meniscus was 0.9mm (range, -1.9 to 3.4).

In the MMRT group, three patients had anterior cruciate ligament (ACL) tears and one patient had a posterior cruciate ligament (PCL) tear. Five patients had a history of trauma whereas 17 patients had not experienced a traumatic event. However, 19 patients had ACL tear sand none of them had PCL tears in the LMRT group. Nineteen patients had a history of trauma. In the MMRT group, fourteen patients showed K-L grade 3 and 1 patient showed K-L grade 4 osteoarthritis. In the LMRT group, 14 patients did not show osteoarthritic change in the knee x-rays with K-L grade 0~1. Only 4 patients showed K-L grade 2 ad 2 patients showed 3 osteoarthritis.

**Fig 2:**
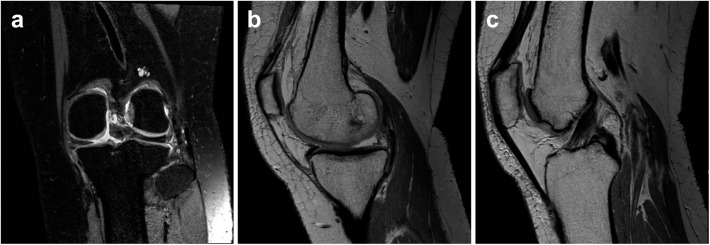
Knee MRI of a patient who had a medial meniscus root tear. Fat-saturated proton-density-weighted images in the coronal scan (a) and proton-density-weighted sagittal (b) scan showed a defect in the medial meniscus posterior horn root. A medial extrusion of the meniscus and high-grade chondral lesions due to osteoarthritis are noted. A proton-density-weighted sagittal scan at a different level (c) showed an ACL that was intact.

Fig 2 is an MRI of a 55-year-old female patient who had difficulty standing, walking up the stairs, and squatting for five months. She did not have a history of trauma. Her left knee MRI revealed a medial meniscus root tear with medial extrusion. The size of the extrusion was 5.4 mm. The ACL was intact. She underwent an arthroscopic pull-out suture for her medial meniscus posterior horn root tear.

**Fig 3:**
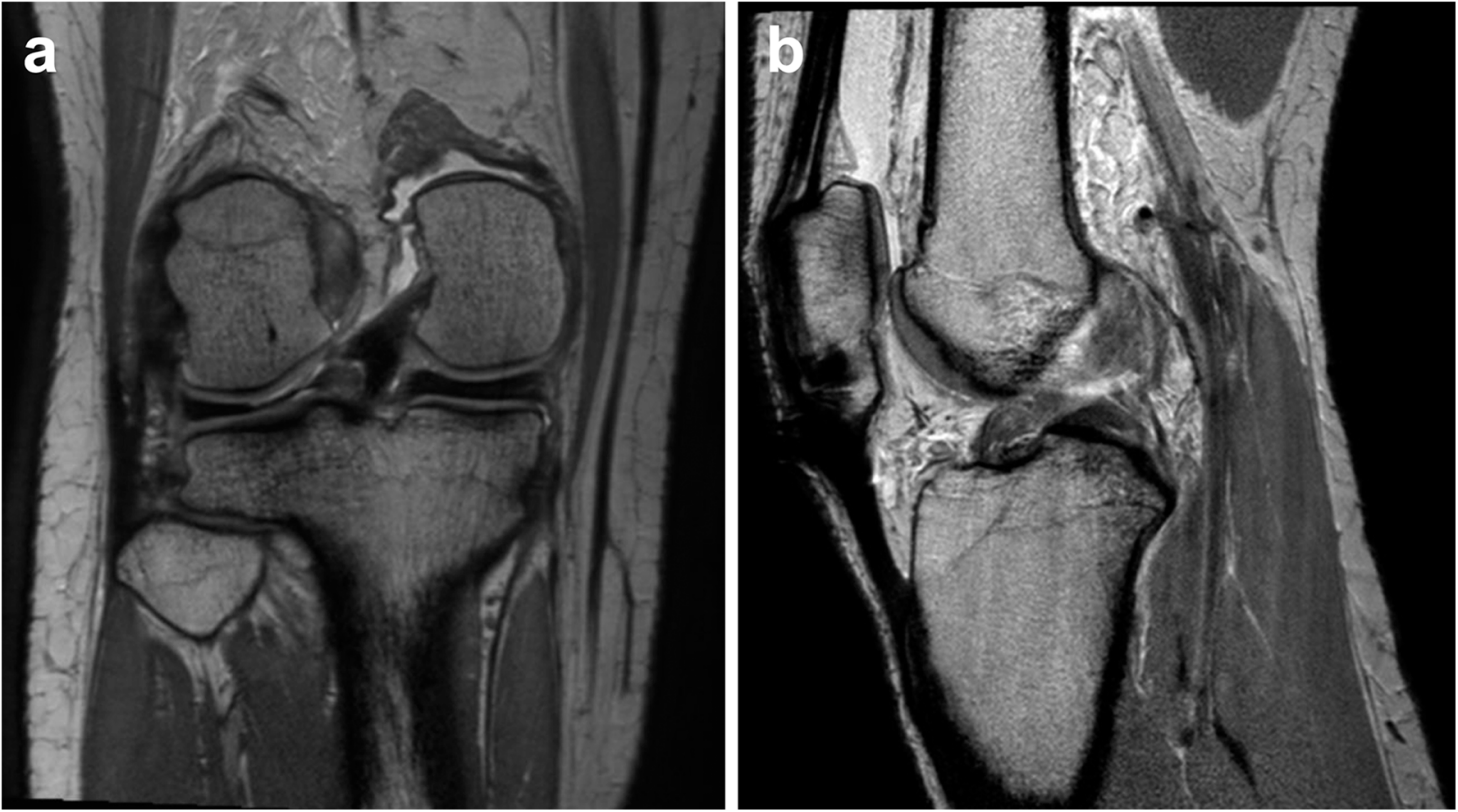
Knee MRI of a patient who had a lateral meniscus root tear. Proton-density-weighted coronal scan (a) showed increased signal intensity on the lateral meniscal root, suggesting a meniscal tear. Although there was a meniscal root tear, meniscal extrusion was not evident. In addition, there was an anterior cruciate ligament tear. Focal discontinuity in the mid-segment of the anterior cruciate ligament with diffuse swelling was seen in the proton-density-weighted sagittal scan (b).

A twenty-year-old female handball player suffered a traumatic injury during exercise. She slipped down onto her right knee and heard a popping sound. After the injury, she couldn’t flex the knee and felt unstable. MRI (Fig 3) of her knee was taken 10 days after the injury. It showed an ACL rupture and lateral meniscus root tear. Although there was a lateral meniscus root tear, it was not accompanied by meniscal extrusion, measuring 0.3mm from the tibial plateau. She underwent arthroscopic ACL reconstruction and lateral meniscus root repair.

There was a significant correlation between MMRT and meniscal extrusion (*p* < 0.0001), and between an ACL tear and LMRT (*p* < 0.0001). A history of trauma was significantly common in LMRTs (*p* < 0.0001). LMRT patients were significantly younger than MMRT patients (*p* < 0.0001). K-L grade differed significantly between the groups (*p* < 0.0001). In terms of the K-L grade, MMRT group had osteoarthritis more frequently than LMRT group. However, there was no correlation between the tear site and the knee joint whether it was left or right (*p* = 0.0654). Table 1 summarizes the results.

**Table 1:** Comparison between a medial meniscus root tear and lateral meniscus root tear.

	Medial meniscus root tear	Lateral meniscus root tear	*p*-value
**Number of patients in study group**	22	20	
**Mean age of patients (years)**	52 (range, 29 ~ 71)	30 (range, 14 ~ 62)	*p*<0.0001
**Meniscal extrusion**			
**Number of meniscal extrusion case**	18	1	*p*<0.0001
**Mean extent of meniscal extrusion (mm)**	4.2 (range, 0.6 ~ 7.8)	0.9 (range, -1.9 ~ 3.4)	
**Number of ACL tear case**	3	19	*p*<0.0001
**Number of patients who had trauma**	5	19	*p*<0.0001
**Kellgren-Lawrence grade**			
** Grade 0~1**	3	14	*p*<0.0001
** Grade 2**	4	4	
** Grade 3**	14	2	
** Grade 4**	1	0	

In the multi-variable analysis using logistic regression, meniscal extrusion was less likely to be accompanied by LMRT (*p* = 0.0032, odds ratio: 0.045, 95% CI: 0.003–0.773). In addition, there was a significant correlation between an LMRT and ACL tear (*p* = 0.0227, odds ratio: 45.867, 95% CI: 1.706 - >999.999).

## Discussion

We assumed that MRI results and clinical findings differ between medial and lateral meniscus root tears. Our study result showed that meniscal extrusion was more prevalent in MMRT patients. ACL tear and trauma history was significantly common in LMRT patients. LMRT patients were younger than MMRT patients.

Various structures provide direct and indirect attachments of the meniscus to the femur and tibia to maintain its optimal position. Among them, direct fixation of the menisci to the central tibial plateau by meniscal roots is critical for the biomechanical functioning of the knee joint. The meniscal roots help to resist hoop stress during axial loading [1]. In a meniscus root tear, this function is disrupted and radial expansion and displacement of the meniscus from the joint space may occur. Then, the axial compressive forces on the knee are transmitted directly to the articular cartilage, resulting in cartilage degeneration and osteoarthritis [2, 6–10].

The peripheral borders of the medial and lateral menisci are indirect attachments of the meniscus to the femur and tibia. As such, those peripheral borders are attached to the fibrous joint capsule except by the tendon sheath of the popliteal tendon, where the lateral attachment of the meniscus to the capsule is interrupted. The medial meniscus is firmly attached to the joint capsule and the deep medial collateral ligament. However, the lateral meniscus has no attachment to the lateral collateral ligament. The medial meniscus is also fixed to the inferior margin of the tibial plateau by the coronary ligament. On the other hand, the lateral meniscus is attached to the joint capsule by the meniscofemoral ligaments, popliteal tendon, popliteo-meniscal fascicle, and arcuate complex. The menisci are attached to each other anteriorly by the transverse ligament [1, 2, 11].

The capsular attachment of the medial meniscus is tighter than that of the lateral meniscus. Therefore, when the medial capsule is displaced by fluid or osteophytes, the attached medial meniscus is pulled with it. However, joint fluid tends to collect in the relatively lax lateral meniscus, yielding menisco-synovial recesses rather than displacing the lateral meniscus [17, 18]. These anatomic differences between the lateral and medial menisci explain our study results that meniscus extrusion was more prevalent in medial meniscus root tears.

Similar to the previous study results, our study demonstrated that lateral meniscus root tears were more prevalent than medial meniscus root tears in patients with ACL tears [11]. The possible mechanism causing lateral meniscus root tear during ACL injury is thought to be the impingement of relatively fixed bony insertion site of lateral meniscus during knee subluxation. In addition, we think characteristic anatomic features may partially explain the accompanying posterior root tear. The anterior root of the lateral meniscus inserts into the tibia, where it partially blends with the ACL. The fibers of the anterior root of the lateral meniscus intermingle with anterior and lateral fibers of the ACL. Because of the shared tibial insertion site of both the ACL and anterior root of the lateral meniscus, a relatively weak posterior root without other intermingling fibers may be torn after ACL injury [1].

It is understandable that medial meniscus root tears are usually degenerative. Because of its looser connections to the capsule and ligaments, the lateral meniscus is more mobile than the medial meniscus. Therefore, the lateral meniscus is theoretically more resistant to weight bearing force,. However, further investigations are needed to clearly understand the mechanism of a traumatic lateral meniscus root tear and its relationship to an ACL tear.

Because the posterior horn is not easily visualized arthroscopically, these tears may be overlooked unless the examiner specifically searches for them. Therefore, an accurate MRI diagnosis of a posterior root tear of the meniscus is clinically important. However, it can be difficult to diagnose a posterior root tear of the meniscus based on MRI due to the pulsation artifact from the popliteal artery, complex anatomy related to the origin of the meniscofemoral ligament, volume averaging and magic angle effect because of the slope of the meniscus on the tibial eminence [14, 15]. Therefore, the meniscal extrusion on MRI should be used as an additional clue for diagnosing a meniscal root tear, especially in the case of a medial meniscus root tear. Also, the lateral meniscus should be carefully evaluated when there is an ACL tear even though meniscal extrusion is rare in a lateral meniscus root tear. A different approach to reading MRI is recommended when diagnosing and evaluating medial and lateral meniscus root tears.

Our study has several limitations. Firstly, this was a retrospective study, and a selection bias exists because cases were selected on the basis of the presence of some degree of meniscus root tears. In addition, our study only included patients with meniscal tears that had been confirmed arthroscopically. Furthermore, the presence of meniscal tears are not only seen in MRI, but also confirmed through the operation. Although this can be considered an advantage of our study, it can also be a limitation. Many patients with a MMRT undergo medical treatment instead of an operation because they have osteoarthritis at the same time and they don’t usually require a further arthroscopic procedure. Therefore, if we included those patients who do not have arthroscopic confirmation in the study population, the results may have differed. Secondly, a supine MRI may inadequately reveal the true amount of extrusion occurring in weight-bearing conditions. Thirdly, the sample size of the root tear group was small. As mentioned above, the sample size was small because we only included the confirmed cases. Fourthly, time interval from injury to MRI may affect the degree of meniscal extrusion. Even though only a small number of MMRT patients experiences significant traumas, many of them may have experienced some episodes with minor trivial but shock-like pains. So we tried to analyze the correlation between injury time and meniscal extrusion of MMRT patients. However, several patients didn’t remember the time of injury or pain exactly. Furthermore, even if they remember the exact time, many of them didn’t visit the hospital or take the MRI evaluation immediately. Therefore, we found it difficult to analyze the correlation between injury time and meniscal extrusion using our data.

## Conclusion

In conclusion, meniscal extrusion is a common finding in patients with a medial meniscus root tear. However, it is rare in patients with a lateral meniscus root tear, which is more commonly associated with a history of trauma and an ACL tear.
